# Mucocele

**Published:** 1892-04-23

**Authors:** 


### OPHTHALMOLOGY.

### MUCOCELE.

Mucocele is the result of distension of the lachrymal sac with chronic thickening of the mucous membrane which lines it; the mucous membrane pours out an increased secretion, which at first only mucous, may become more turbid and eventually purulent.

It is but rarely a primary affection, being generally the result af some obstruction to the outflow of the secretion, from the lachrymal sac, or to increased secretion. Obstruction may occur at either end of the sac, and may be caused by occluded punctum lachrymalis, stricture of the canaliculus, dacroliths, or fungi, or even polypi, diseases of the bones or injuries to the surrounding parts. Epiphora is likely to be one of the earlier symptoms; as we saw in a previous number of the The Hospital it was generally a symptom of obstruction in these parts.

A little swelling first appears at the inner canthus, and fluctuates under the finger when pressed upon, mucous secretion being forced by the pressure out of the punctum, or down into the nose. If this goes on without appropriate treatment the sac may become inflamed, in which case the skin becomes stretched, is reddish-brown in appearance, and hard and brawny to the touch, the secretion becomes purulent, and a lachrymal abscess is formed with a tendency to point outwards. If this burst of its own accord a troublesome fistula may be formed. When an abscess has formed there is much pain, and sometimes irritation of the whole system; the brawny condition of the skin is often mistaken for erysipelas, and patients will often say it began with that disease. The pus not infrequently burrows among the surrounding cellular tissue.

In the treatment of mucocele the first principle is to remove the obstruction. If the punctum be occluded, an artificial one must be made; if stricture be the cause, gradual dilatation by probing must be accomplished, and if the stricture be very tough it can be divided by cutting with a Weber's canaliculus knife. The inflamed or thickened mucous membrane must also be treated by the use of mild astringent applications. Professor Meyer's plan is as follows:

Whether the patient comes with the disease commencing or advanced, he first ascertains whether a stricture exist. He introduces the nozzle of his lachrymal syringe, in which is contained a 10 grs. to the ℥i solution of hydrochlorate of cocain, through the punctum and canaliculus into the lachrymal sac; if the punctum is small he dilates it with graduated probes. After the cocain has been injected into the sac he observes if it pass down into the nose or regurgitate through the punctum on the upper lid, if the latter it shows that a stricture of the nasal duct exists, which will require probing. The cocain solution, besides this use, subdues to some extent the pain that introduction of a probe causes, and which is generally very severe. He then washes out the sac with an astringent solution, such as zinc sulphate, of the strength of one or two grains to the ounce of water, and continues this daily until only clear mucous escapes, preceding it by the probing, and this treatment is found to be the most successful.

When a patient cannot be seen regularly he should have an astringent lotion to use at home by dropping some into the conjunctional sac, when some of the solution will find its way through the lachrymal canal. When treatment of this kind fails, the wearing of a style, a kind of probe, may be recommended. Hollow ones are made through which the tears can flow; they should be made of silver, and furnished with a rim or head, lest they slip out of reach.

Removal of the lachrymal gland is sometimes practised in cases of chronic abscess, and the sac in similar cases has been obliterated by the actual cautery.

The cure of fistula must be preceded by re-establishing the lachrymal passage, introducing a hollow style, and then paring the edges of the fistula wound, and stitching them together. It will, however, generally be found an obstinate complaint to treat, as, even when wearing the hollow style, some tears escape the wrong way, and being saline, keep up irritation in the wound.

